# Impact of *HFE* variants and sex in lung cancer

**DOI:** 10.1371/journal.pone.0226821

**Published:** 2019-12-19

**Authors:** Sang Y. Lee, Vonn Walter, Junjia Zhu, Anna C. Salzberg, Dajiang J. Liu, James R. Connor

**Affiliations:** 1 Department of Neurosurgery, The Pennsylvania State University College of Medicine, Penn State Health Milton S. Hershey Medical Center, Hershey, Pennsylvania, United States of America; 2 Department of Public Health Sciences, The Pennsylvania State University College of Medicine, Penn State Health Milton S. Hershey Medical Center, Hershey, Pennsylvania, United States of America; 3 Department of Biochemistry and Molecular Biology, The Pennsylvania State University College of Medicine, Penn State Health Milton S. Hershey Medical Center, Hershey, Pennsylvania, United States of America; 4 Institute for Personalized Medicine, The Pennsylvania State University College of Medicine, Penn State Health Milton S. Hershey Medical Center, Hershey, Pennsylvania, United States of America; Seoul National University College of Pharmacy, REPUBLIC OF KOREA

## Abstract

The homeostatic iron regulator protein HFE is involved in regulation of iron acquisition for cells. The prevalence of two common *HFE* gene variants (H63D, C282Y) has been studied in many cancer types; however, the impact of *HFE* variants, sex and *HFE* gene expression in lung cancer has not been studied. We determined the prevalence of *HFE* variants and their impact on cancer phenotypes in lung cancer cell lines, in lung cancer patient specimens, and using The Cancer Genome Atlas (TCGA) database. We found that seven out of ten human lung cancer cell lines carry the H63D or C282Y *HFE* variant. Analysis of lung cancer specimens from our institute (Penn State Hershey Medical Center) revealed a sex and genotype interaction risk for metastasis in lung adenocarcinoma (LUAD) patients; H63D *HFE* is associated with less metastasis in males compared to wild type (WT) *HFE*; however, females with the H63D *HFE* variant tend to develop more metastatic tumors than WT female patients. In the TCGA LUAD dataset, the H63D *HFE* variant was associated with poorer survival in females compared to females with WT *HFE*. The frequency of C282Y *HFE* is higher in female lung squamous cell carcinoma (LUSC) patients of TCGA than males, however the C282Y *HFE* variant did not impact the survival of LUSC patients. In the TCGA LUSC dataset, C282Y *HFE* patients (especially females) had poorer survival than WT *HFE* patients. *HFE* expression level was not affected by *HFE* genotype status and did not impact patient’s survival, regardless of sex. In summary, these data suggest that there is a sexually dimorphic effect of *HFE* polymorphisms in the survival and metastatic disease in lung cancer.

## Introduction

Lung cancer is the second most common cancer in both men and women and is the leading cause of cancer deaths in the US [1]. There are two types of lung cancers: non-small cell lung cancer (NSCLC) and small cell lung cancer (SCLC). NSCLC is the most common type of lung cancer and accounts for approximately 80–85% of lung cancers [2–4]. There are 3 main types of NSCLC: adenocarcinoma, squamous cell carcinoma, and large cell carcinoma. Adenocarcinoma, the most common form of lung cancer in non-smokers, accounts for about 40% of all lung cancers. Squamous cell carcinoma accounts for roughly 30% and large cell carcinoma is less than 10%. SCLC accounts for ~20% of all lung cancers, with a 5-year survival of only 5–10% [4, 5]. Molecular profiling of NSCLC found higher gene mutations in multiple oncogenes, such as epidermal growth factor receptor (*EGFR*) (10–35%), K-ras (*KRAS*) (15–25%), and Phosphatase and tensin homolog (*PTEN*) (4–8%). Other gene alterations, e.g. Fibroblast growth factor receptor 1 (*FGFR1*) amplification (20%) and anaplastic lymphoma kinase (*ALK*) rearrangement (3–7%) are also found in NSCLC [6–8]. Interestingly, many driver mutations found in lung adenocarcinoma (LUAD) are rarely found in lung squamous cell carcinoma (LUSC), which suggests distinct cancer development processes among lung cancer sub-types.

Cancer cells have a robust iron appetite associated with their higher growth and metabolism [9]. Iron can influence the epigenetics of cancer cells [10] and the tumor microenvironment by impacting macrophage function [11, 12]. Among the many iron metabolism genes, the *HFE* (homeostatic iron regulator) gene has been interrogated for its relationship to cancer. The *HFE* gene encodes for a 343-amino acid major histocompatibility complex (MHC) class 1 molecule [13] whose interaction with the transferrin receptor (TFRC) on the cell membrane regulates the amount of iron internalized to the cell by limiting the interaction of transferrin (TF) with the TFRC. The *HFE* gene contains genetic polymorphisms identified as risk factors or disease modifiers for several human diseases such as hereditary hemochromatosis, neurodegenerative diseases, liver disease, and cancers [14–18].

Polymorphisms in the *HFE* gene occur more frequently in Caucasians than in other races [19–21]. There are two major mutation sites in the *HFE* gene [13]. One is H63D, a single mutation of C to G at nucleotide 187, which results in a substitution of aspartate for histidine at amino acid 63. The other one is C282Y, a mutation of G to A at nucleotide 845, which results in the substitution of tyrosine for cysteine at amino acid 282. In the normal Caucasian population, the frequency of H63D and C282Y *HFE* genotype is around 26% (24% heterozygote and 2.4% homozygote) and 10% (10% heterozygote and 0.44% homozygote), respectively [19]. The allelic frequency of H63D and C282Y *HFE* in the general Caucasian population is 15.3% and 6.8% [20]. Increased frequency of the *HFE* variants have been reported in acute lymphoblastic leukemia [22, 23] and breast cancer [24, 25]. Increased frequency of H63D *HFE* variant was observed in malignant gliomas [26]. The H63D *HFE* variant is also a risk factor for colorectal cancer [27], hepatocellular carcinoma [28–30], and pancreatic cancer [30, 31]. Increased frequency of C282Y *HFE* variant was observed in colorectal cancer [32] and hepatocellular carcinoma [33]. In addition, the C282Y *HFE* variant is a risk factor for breast cancer [31, 34, 35], colorectal cancer [34, 35], hepatocellular carcinoma [30, 31, 35, 36], liver cancer [37], and ovarian cancer [38]. The risk of C282Y *HFE* variant in colorectal cancer has been reported as both increased [32] and decreased [31]. In our previous study using Penn State Health Milton S. Hershey Medical Center (PSHMC) glioblastoma (GBM) patient’s samples, we demonstrated poorer overall survival in male GBM patients with H63D *HFE* than male WT *HFE* GBM patients [39] although this finding was not observed in TCGA GBM samples [40].

Despite the prevalence of lung cancer and the prevalence of the *HFE* gene variants, the impact of *HFE* genotype on lung cancer has not been studied systematically and this knowledge gap is addressed in this study. We interrogated the *HFE* genotype and/or *HFE* expression in human lung cancer cell lines, specimens of lung cancer patients, and lung cancer database.

## Materials and methods

### Cell culture

All tested lung cancer cell lines were ordered from American Type Culture Collection (ATCC, Manassas, VA) or obtained from Dr. Jong K. Yun (Pennsylvania State University College of Medicine). Human lung cancer cell lines were maintained in Roswell Park Memorial Institute (RPMI) media 1640 with 1X Penicillin-Streptomycin and 10% fetal bovine serum (FBS). RPMI media 1640 and other cell culture ingredients were purchased from Life Technologies (Grand Island, NY).

### *HFE* genotype of cell lines and plasma of lung cancer patients at the Penn State Health Milton S. Hershey Medical Center (PSHMC)

*HFE* genotype of lung cancer cell lines was determined using a restriction enzyme digestion method after PCR. The digested PCR products were run in 5% TBE polyacrylamide gel for *HFE* genotype as reported [41]. The *HFE* genotyping from plasma of lung cancer patients was performed using same method after genomic DNA purification using DNeasy Tissue kit (Qiagen) according to the manufacturer's instructions. The *HFE* genotype of select samples was confirmed via DNA sequencing.

The de-identified plasma samples and clinical data of human lung cancer patients were obtained from Tumor Bank of Penn State Institute for Personalized Medicine (PSIPM) and approved by Penn State College of Medicine Institutional Review Board (IRB Protocol Number 40532). DNA was purified from the plasma samples by DNeasy Blood & Tissue kit (QIAAGEN) to determine *HFE* genotype as described above. For *HFE* genotype data analysis, we used only Caucasian lung cancer patients’ samples, because *HFE* polymorphisms are most prevalent in the Caucasian population. We consented and enrolled 53 adenocarcinoma of the lung (23 male and 30 female) and 41 squamous cell lung cancer patients (28 male and 13 female).

The *HFE* genotype of lung cancer patients was compared with samples from individuals without cancer or with neurological disease in our Institute of Personalized Medicine [41] and 1000Genome data which was downloaded the frequency of Single Nucleotide Variants (SNVs) in the *HFE* gene using Variant Call Format (VCF) file from The International Genome Sample Resource.

### The Cancer Genome Atlas (TCGA) lung cancer patient’s data

There are two types of lung cancer data in the TCGA database: lung adenocarcinoma (LUAD) and lung squamous cell carcinoma (LUSC). In the TCGA lung cancer database, we used two samples data from each lung cancer patients. i.e., Blood Derived Normal (NB), Primary Solid Tumor (TP). The total number of NB samples of LUAD patients with corresponding *HFE* genotype information was 408. Among 408 samples, there were 307 Caucasian, 36 Black, 6 Asian, and 59 unknown. The total number of TP samples of LUAD patients who had *HFE* genotype information was 558. Among the 558 samples, there were 381 Caucasian, 52 Black, 8 Asian, 1 American Indian, and 116 unknown. The total number of NB samples of LUSC patients who had *HFE* genotype information was 322. Among 322 samples, there were 203 Caucasian, 15 Black, 6 Asian, and 98 unknown. The total number of TP samples of LUSC patients who had *HFE* genotype information was 507. Among 507 samples, there were 300 Caucasian, 28 Black, 9 Asian, and 170 unknown. We accessed *HFE* gene variant data from Cancer Genomics Hub (CGHub) using GeneTorrent and GTFuse software (AnnaiSystems, Carlsbad, CA) to extract and download only *HFE* genes from complete mapped sequence (BAM) files. Genome Analysis Toolkit (GATK) software, based on the GATK best practices pipeline, was used to identify *HFE* gene variants (H63D, C282Y) from the sequences as we previously reported [40]. Clinical data for the TCGA lung adenocarcinoma and lung squamous cell carcinoma cohorts was accessed with TCGA biolinks [42–44]. During our TCGA lung cancer data analysis, we noticed that some patients had extremely short follow-up times (< 30 days). Because we were concerned that these short survival times could bias the results of our survival analyses, we performed *HFE* genotype status and survival analyses using either (i) all subjects, or (ii) all subjects with follow-up times greater than 30 days (both living and deceased). The two approaches produced highly concordant results. Therefore, the results presented here are based on an analysis of all patients.

### Association between *HFE* genotype/*HFE* expression/Sex and survival in TCGA lung cancer patients

RNA sequencing (RNA-Seq) and clinical data from the TCGA LUAD and LUSC cohorts were accessed from the Broad Institute’s Firehose GDAC (https://gdac.broadinstitute.org/). We applied a log transformation to the resulting normalized RSEM (RNA-Seq by Expectation-Maximization) values from RNA-Seq, so gene expression was quantified as log2 (RSEM + 1). Primary tumor samples (LUAD, n = 515; LUSC, n = 445) and matched patient’s normal samples (LUAD, n = 58; LUSC, n = 33) were identified from the TCGA barcodes. Kruskal-Wallis tests or Wilcoxon rank sum tests were used to compare *HFE* expression values in the following groups: (i) *HFE* variant vs. *HFE* wild type (WT), (ii) females vs. males. Separate analyses were performed for primary tumor and matched blood samples in each subject. Additionally, H63D and C282Y variants were considered separately in each tumor type, and the variant status for matched normal samples was determined by the status of the matched primary tumor sample. Kaplan-Meier plots and log rank tests were applied to compare survival times in groups defined by *HFE* expression level. Survival times were compared for females and males after defining *HFE* expression groups as high or low based on median expression or based on quartiles. Additionally, multivariable Cox proportional hazards models were fit using either *HFE* genotype or *HFE* expression while adjusting for tumor stage (early stage (I/II) vs. advanced stage (III/IV)), age at diagnosis, gender, and smoking (never smoked vs. any smoking history). All survival analyses were performed after restricting to Caucasians only.

### Statistical analysis

Comparisons of *HFE* gene variant frequency between samples were determined using Fisher‘s exact test or Chi-square test. Additionally, the associations between *HFE* gene variant and sex were examined using Fisher’s exact test. The association between patients’ overall survival and *HFE* polymorphisms (H63D, C282Y) was indicated by Kaplan-Meier survival curve and analyzed by log-rank test. The log-rank test results were further examined using multi-variable Cox proportional hazards regression models, by controlling for several known confounders. R 3.5.0 (R Foundations) was used to conduct all data analyses for *HFE* expression related study [45], and the survival analyses were performed using the survival R package [46] and GraphPad Prism 7 software. All tests were two-sided and the statistical significance level was set to 0.05.

## Results

### *HFE* genotype of lung cancer cell lines

Of the ten lung cancer cell lines investigated, 6 expressed the H63D *HFE* variant and 1 had the C282Y *HFE* variant (**Table 1**). Only 1 large cell lung cancer cell line did not have a *HFE* gene variant and the only squamous cell carcinoma cell line was heterozygote for C282Y.

**Table 1:** *HFE* genotype of lung cancer cell lines.

Name	Tissue or cancer type	Stage	*H63D HFE*	*C282Y HFE*
A549	Carcinoma	NA	*hetero*	wildtype
H23	Adenocarcinoma, Non-small cell lung cancer	NA	*hetero*	wildtype
H441	Papillary adenocarcinoma	NA	*hetero*	wildtype
H460	Carcinoma; large cell lung cancer	NA	wildtype	wildtype
H520	Squamous cell carcinoma	NA	wildtype	*hetero*
H522	Adenocarcinoma, Non-small cell lung cancer	2	*hetero*	wildtype
H838	Adenocarcinoma, Non-small cell lung cancer	3B	wildtype	wildtype
H1299	Carcinoma, Non-small cell lung cancer	NA	wildtype	wildtype
H1650	Adenocarcinoma; bronchoalveolar carcinoma	3B	*hetero*	wildtype
H1993	Lung adenocarcinoma	3A	*homo*	wildtype

NA (not available); hetero (heterozygote); homo (homozygote)

### Frequency of *HFE* genotype in lung cancer patients

We determined the frequency of *HFE* gene variants in lung cancer patients (adenocarcinoma of the lung, squamous cell lung cancer) seen in our Penn State Hershey Medical Center (PSHMC), and publically available lung cancer databases. The characteristics of lung cancer patients, such as median age, age range, and male: female ratio, are summarized in **Table 2**.

**Table 2:** Characteristics of our study sample, TCGA lung cancer, and 1000Genome data.

	PSHMC LUAD (n = 53)	PSHMC LUSC (n = 41)	TCGA LUAD_NB (n = 307)	TCGA LUAD_TP (n = 381)	TCGA LUSC_NB (n = 203)	TCGA LUSC_TP (n = 300)	PSHMC Non-cancer (n = 94) ^a^	White in 1000Genome Phase 3 (n = 185) ^b^
Median age (years old)	70, (Male: 71; Female: 68.5)	73, (Male: 73; Female: 70)	67, (Male: 66.5; Female: 67)	67, (Male: 66.5; Female: 67)	68, (Male: 68; Female: 69.5)	68, (Male: 68; Female: 69)	58, (Male: 61; Female: 56)	NA
Range of age (years old)	48–90, (Male: 54–84; Female: 48–90)	40–84, (Male: 54–81; Female: 40–84)	33–88, (Male: 38–88; Female: 33–87)	33–88, (Male: 38–88; Female: 33–87)	44–85, (Male: 46–85; Female: 44–83)	40–85, (Male: 40–85; Female: 44–83)	40–85, (Male: 41–82; Female: 40–85)	NA
Male : Female (ratio)	23 : 30 (1 : 1.3)	28 : 13 (2.15: 1)	140 : 167 (1 : 1.2)	172 : 209 (1 : 1.2)	151 : 52 (2.9 : 1)	219 : 81 (2.7 : 1)	34 : 60 (1 : 1.8)	80 : 102 (1 : 1.3)

LUAD (lung adenocarcinoma)

LUSC (lung squamous cell carcinoma)

NB (blood normal)

TP (tumor patient)

^**a**^Lee SY et al. PLoS One. 2017;12(3):e0174778. [40]

^**b**^There are a total of 1,077 samples (527 male, 550 female) listed on the website, however, only a subset have sequences. There are 185 European subpopulation (80 male, 102 female, 3 unknown). Age information is not available.

We limited our analysis to Caucasians because of the higher frequency of *HFE* polymorphisms compared to other races [19–21]. Among 53 lung adenocarcinoma patients in PSHMC, the frequency of H63D and C282Y *HFE* gene variant was 32.0% and 13.2%, respectively (**S1 Table)**. In squamous cell lung cancer patients, the frequency of H63D and C282Y *HFE* variant was 14.6% and 12.2%, respectively (**S1 Table**). The allele frequency of H63D *HFE* was 20.8% and 8.5% for C282Y *HFE* in lung adenocarcinoma, and 7.3% for H63D *HFE* and 7.3% for C282Y *HFE* in lung squamous cell carcinoma. Statistical analysis revealed a lower frequency of H63D *HFE* alleles in squamous cell carcinoma compared to adenocarcinoma (p = 0.007). In comparison to non-cancer population adenocarcinoma tended to have higher C282Y *HFE* allele frequency (p = 0.08) compared to 1000Genome database. Squamous cell carcinoma had significantly lower H63D *HFE* allelic frequency than the PSHMC control samples (p = 0.0336) or 1000Genome data (p = 0.04).

**Fig 1:**
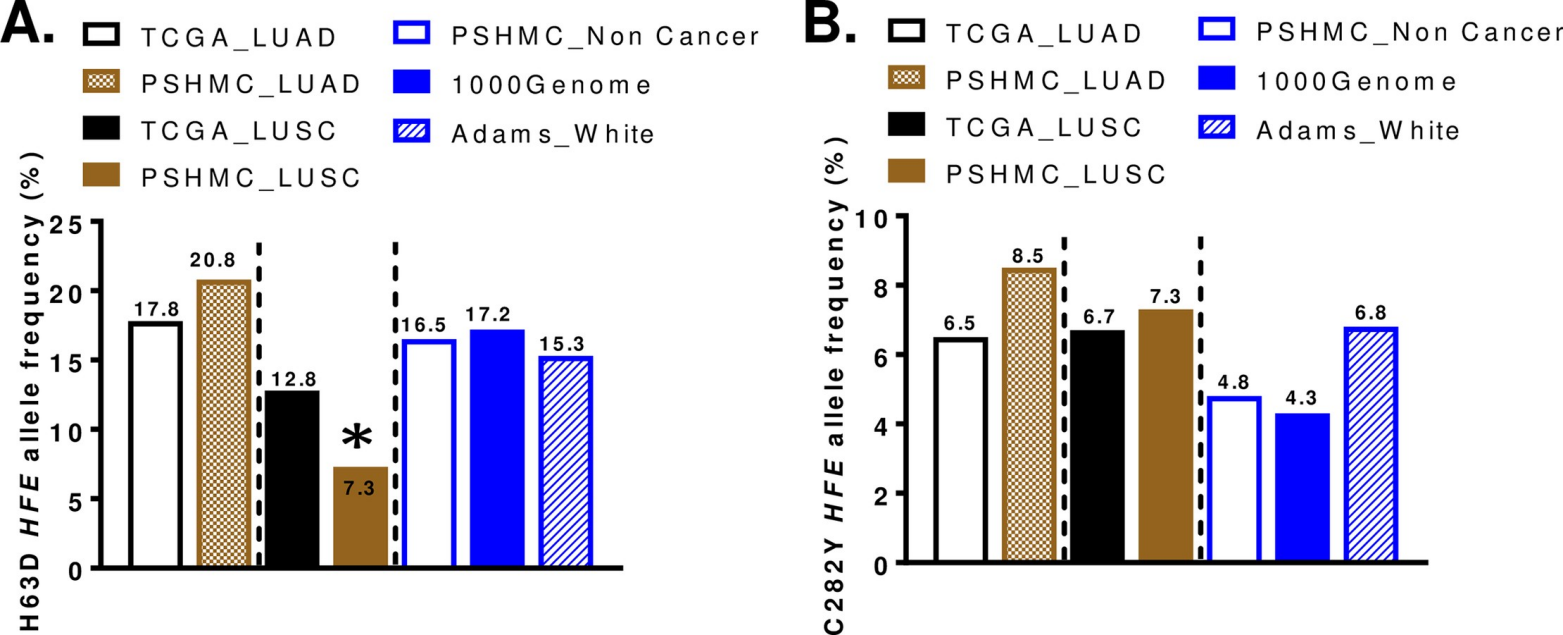
Allele frequency of *HFE* variants in lung cancers and control population (non-cancer general population). (A) H63D *HFE* allele frequency in the blood samples of lung cancer patients (TCGA LUAD or LUSC, PSHMC) and non-cancer control (PSHMC non-cancer, 1000Genome, and White of Adams study in 2005 [20]). The asterisk (*) indicates significantly decreased H63D *HFE* allele frequency in LUSC of PSHMC patients compared to non-cancer control population. p<0.05 (B) C282Y *HFE* allele frequency in the blood samples of lung cancer patients (TCGA LUAD or LUSC, PSHMC) and general population (PSHMC non-cancer, 1000Genome, and White of Adams study in 2005 [20]).

There were no differences between TCGA lung cancer samples (LUAD, LUSC) and non-cancer population for the frequency of *HFE* genotype or *HFE* alleles (**S2 Table**). The summary of allele frequencies for H63D *HFE* and C282Y *HFE* in lung cancers and controls are summarized in **Fig 1**.

### Association between *HFE* genotype and sex in the lung cancer patients

Because distinct male: female ratios exist in two lung cancer types (lung adenocarcinoma, lung squamous cell carcinoma), the *HFE* genotype data were stratified by sex. There was an increase in the C282Y *HFE* allelic frequency in female LUAD patients in the TCGA database (p = 0.0474) (**Table 3**) but not in the PSHMC database (**S3 Table**). There were no sex differences in the H63D *HFE* allele groups.

**Table 3:** Frequency of *HFE* genotype and alleles based on sex in TCGA lung cancer (Caucasian patients).

	TCGA LUAD				TCGA LUSC			
	NB (n = 307)		TP (n = 381)		NB (n = 203)		TP (n = 300)	
	Male (n = 140)	Female (n = 167)	Male (n = 172)	Female (n = 209)	Male (n = 151)	Female (n = 52)	Male (n = 219)	Female (n = 81)
**Genotype**								
*H63/D63* (heterozygote)	37 (26.4%)	46 (27.5%)	41 (23.8%)	57 (27.3%)	32 (21.2%)	13 (25.0%)	46 (21.0%)	20 (24.7%)
*D63/D63* (homozygote)	6 (4.3%)	7 (4.2%)	6 (3.5%)	8 (3.8%)	3 (2.0%)	1 (1.9%)	7 (3.2%)	3 (3.7%)
*C282/Y282* (heterozygote)	12 (8.6%)	22 (13.2%)	14 (8.1%)	30 (14.4%)	20 (13.2%)	5 (9.6%)	27 (12.3%)	5 (6.2%)
*Y282/Y282* (homozygote)	0 (0.0%)	3 (1.8%)	1 (0.6%)	2 (1.0%)	0 (0.0%)	1 (1.9%)	0 (0.0%)	1 (1.2%)
**Alleles**								
*H63D HFE*	49/280 (17.5%)	60/334 (18.0%)	53/344 (15.4%)	73/418 (17.5%)	38/302 (12.6%)	15/104 (14.4%)	60/438 (13.7%)	26/162 (16.1%)
*C282Y HFE*	12/280 (4.3%)	28/334 (8.4%)	16/344 (4.7%)	34/418 (8.1%)	20/302 (6.6%)	7/104 (6.7%)	27/438 (6.2%)	7/162 (4.3%)
**Fisher’s exact test (Male vs. Female)**	p = 0.9142 (H63D) **p = 0.0474 (C282Y)**		p = 0.4268 (H63D) **p = 0.0549 (C282Y)**		p = 0.6082 (H63D) p = 1.0 (C282Y)		p = 0.4249 (H63D) p = 0.4308 (C282Y)	

Values were expressed as n = N (%)

LUAD (lung adenocarcinoma)

LUSC (lung squamous cell carcinoma)

NB (blood normal)

TP (tumor patient)

### Association between *HFE* genotype and *HFE* expression and sex in TCGA lung cancer data (Caucasian patients)

**Fig 2:**
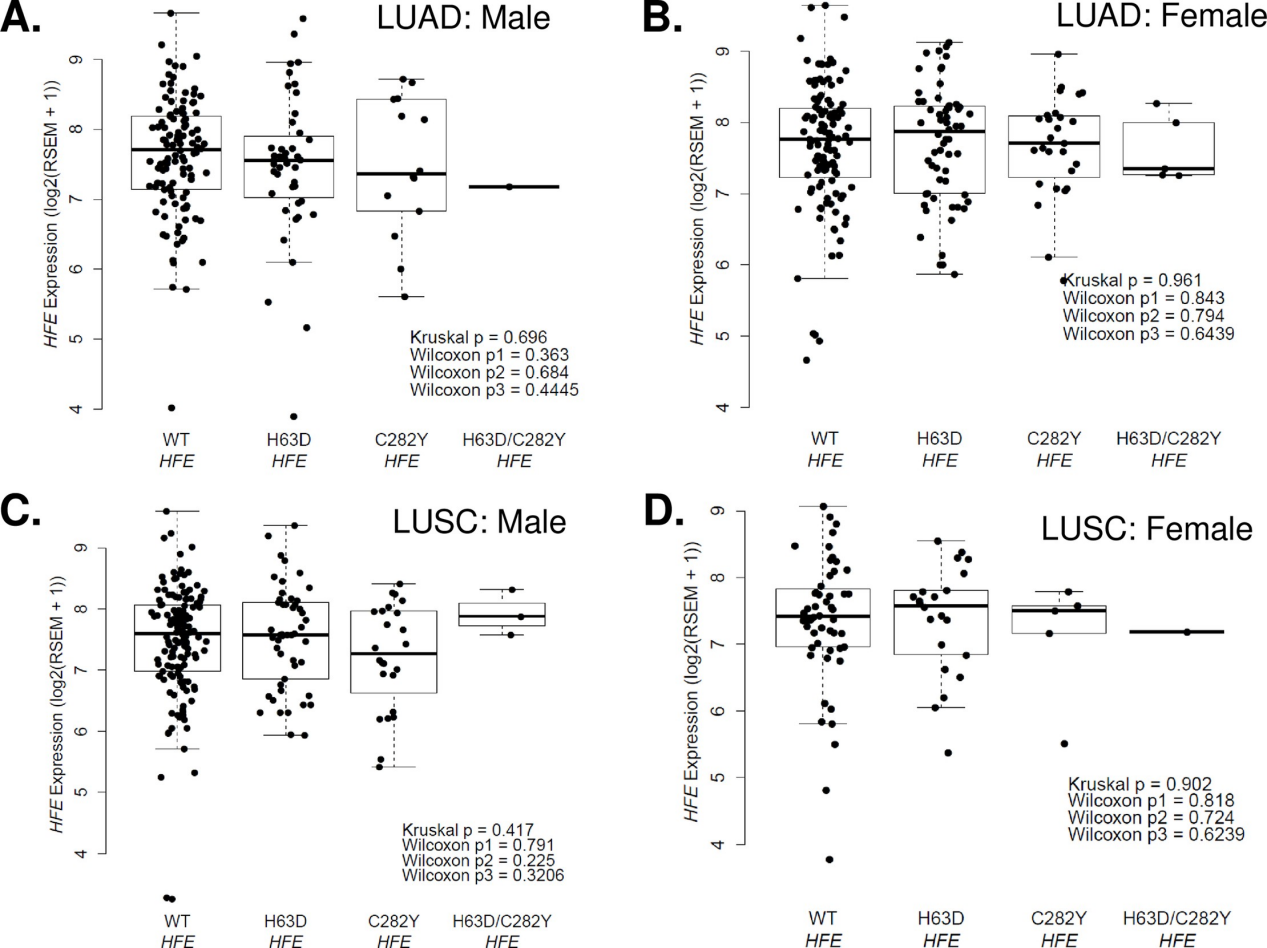
Association between *HFE* expression and *HFE* genotype or sex in TCGA lung cancer data. **(A)** *HFE* gene expression based on *HFE* genotype (WT, H63D, C282Y, H63D and C282Y) in male primary tumor LUAD patients. **(B)** *HFE* gene expression based on *HFE* genotype (WT, H63D, C282Y, H63D and C282Y) in female primary tumor LUAD patients. **(C)** *HFE* gene expression based on *HFE* genotype (WT, H63D, C282Y, H63D and C282Y) in male primary tumor LUSC patients. **(D)** *HFE* gene expression based on *HFE* genotype (WT, H63D, C282Y, H63D and C282Y) in female primary tumor LUSC patients. Kruskal p = the p-value for the Kruskal-Wallis test comparing the median *HFE* expression levels in each of the four groups, Wilcox p1 = the p-value for the Wilcoxon rank sum test comparing the median *HFE* expression levels in WT *HFE* vs. H63D *HFE*, Wilcox p2 = the p-value for the Wilcoxon rank sum test comparing the median *HFE* expression levels in WT *HFE* vs. C282Y *HFE*, Wilcox p3 = the p-value for the Wilcoxon rank sum test comparing the median *HFE* expression levels in WT *HFE* vs. H63D/C282Y *HFE*.

We evaluated the association between *HFE* genotype and *HFE* mRNA expression in TCGA lung cancer data. There were no differences between WT and H63D or C282Y *HFE* variants in matched normal or primary tumors of LUAD and LUSC lung cancer patients in two sample t-test (**S1 and S2 Figs**). The level of *HFE* expression was not different between males and females in matched normal or primary tumors of LUAD and LUSC lung cancer patients in two sample t-test (**S1 and S2 Figs**). There were no *HFE* expression differences between WT and *HFE* variants in male or female LUAD and LUSC in the TCGA lung cancer dataset (**Fig 2**).

### Impact of *HFE* genotype on the metastasis of lung cancer patients at PSHMC

Metastatic disease was only available in the PSHMC database. The incidence of metastasis in lung cancer patients at PSHMC revealed no statistical difference between WT and *HFE* polymorphisms but when we stratified for sex, there were significant differences between WT and *HFE* polymorphisms (**Table 4**). None of the H63D *HFE* male adenocarcinoma patients developed metastases while 50% of WT *HFE* male patients did (p = 0.0189); whereas in females the findings were opposite with H63D *HFE* carriers tending to develop more metastases than WT *HFE* (p = 0.0596). With the C282Y *HFE* variant, male adenocarcinoma patients tend to develop more metastases compared to WT *HFE* male carriers (p = 0.0672) while there is no difference in females. In squamous cell lung cancer, neither of the *HFE* variants (H63D and C282Y) or sex impacted metastatic rate.

**Table 4:** *HFE* genotype and metastasis rate based on sex of lung cancer patients at PSHMC.

***Male***		**PSHMC LUAD (n = 23),** **n (%)**	**Metastasis, n (%)**	**Fisher’s exact test (WT *HFE* vs variant *HFE*)**	**PSHMC LUSC (n = 28), n (%)**	**Metastasis, n (%)**	**Fisher’s exact test (WT *HFE* vs variant *HFE*)**
*H63D HFE*	CC	14 (60.9%)	7 (50.0%)	**p = 0.0189**	24 (85.7%)	10 (41.7%)	p = 0.0978
	C*G* or *GG*	9 (39.1%)	0 (0.0%)		4 (16.7%)	4 (100.0%)	
*C282Y HFE*	GG	19 (82.6%)	4 (21.1%)	p = 0.0672	24 (85.7%)	11 (45.8%)	p = 0.5956
	G*A* or *AA*	4 (17.4%)	3 (75.0%)		4 (16.7%)	3 (75.0%)	
***Female***		**LUAD (n = 30), n (%)**	**Metastasis, n (%)**	**Fisher’s exact test (WT *HFE* vs variant *HFE*)**	**LUSC (n = 13), n (%)**	**Metastasis, n (%)**	**Fisher’s exact test (WT *HFE* vs variant *HFE*)**
*H63D HFE*	CC	22 (73.3%)	3 (13.6%)	**p = 0.0596**	11 (84.6%)	2 (18.2%)	p = 1.0
	C*G* or *GG*	8 (26.7%)	4 (50.0%)		2 (15.4%)	0 (0.0%)	
*C282Y HFE*	GG	27 (90.0%)	7 (25.9%)	p = 1.0	12 (92.3%)	2 (16.7%)	p = 1.0
	G*A* or *AA*	3 (10.0%)	0 (0.0%)		1 (7.7%)	0 (0.0%)	

LUAD (lung adenocarcinoma)

LUSC (lung squamous cell carcinoma)

### Association between *HFE* genotype/*HFE* expression/Sex and patient survival in lung cancer patients at PSHMC and TCGA lung cancer data

There was no survival difference between WT and H63D *HFE* (p = 0.2866 for LUAD; p = 0.3740 for LUSC), or WT and C282Y *HFE* (p = 0.6568 for LUAD; p = 0.2498 for LUSC) lung cancer patients who enrolled at PSHMC (**S3 Fig**).

**Fig 3:**
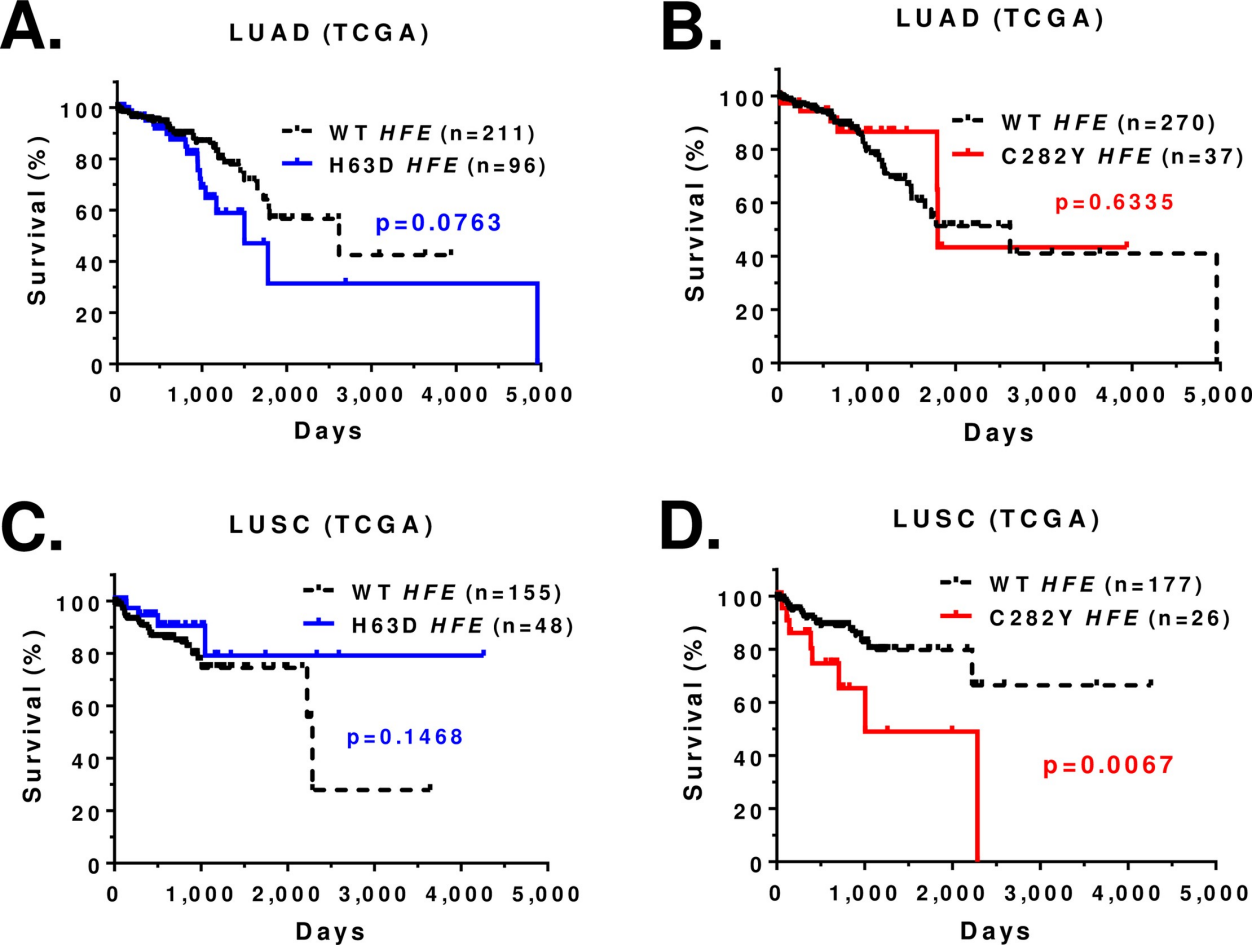
Kaplan-Meier survival curve for TCGA lung cancer patients (Caucasians) with *HFE* genotype. **(A)** Survival curve of LUAD patients with WT or H63D *HFE*. **(B)** Survival curve of LUAD patients with WT or C282Y *HFE*. **(C)** Survival curve of LUSC patients with WT or H63D *HFE*. **(D)** Survival curve of LUSC patients with WT or C282Y *HFE*. Statistical analysis was performed by log-rank test and indicated as p value. Censored record is indicated as + in the graph.

In TCGA database, the Kaplan-Meier survival curve for lung adenocarcinoma patients showed a trend toward poorer survival in H63D *HFE* variants compared to WT *HFE* patients (p = 0.0763) (**Fig 3A**). When the analysis focused on H63D homozygotes compared to WT *HFE* patients, the survival difference was marginally significant (p = 0.0539) (**S4A Fig**). The survival for WT *HFE* patients and C282Y *HFE* patients was not different (**Fig 3B**). The Kaplan-Meier survival curve for LUSC patients revealed a significant survival difference between WT *HFE* patients and C282Y *HFE* patients (p = 0.0067), but not between WT *HFE* and H63D *HFE* variants (**Fig 3C and 3D**).

We performed multiplex Cox regression to double check the bivariate associations (Kaplan-Meier curve) between *HFE* gene variant and overall survival by further controlling other possible factors (tumor stage etc.) that could contribute to the overall survival. The effect of C282Y *HFE* variant on LUSC patient survival remains significant after controlling for age, tumor stage, and smoking status in multiple Cox regression (**Table 5**).

**Table 5:** Hazard ratios from the multiple-variable Cox model for the TCGA lung cancer (Caucasian patients).

	Multivariate analysis			Multivariate analysis	
	HR	P-value		HR	P-value
**LUAD:**					
*H63D variant*	0.71 (0.38–1.32)	0.2803	C282Y *variant*	1.70 (0.70–4.23)	0.2409
*AgeAtDiagnosis*	1.00 (0.98–1.04)	0.5381	*AgeAtDiagnosis*	1.00 (0.98–1.05)	0.4285
*Tumor stage*	3.60 (1.93–6.55)	***<0*.*0001***	*Tumor stage*	4.20 (2.26–7.63)	***<0*.*0001***
*Smoking*	2.00 (1.08–3.55)	***0*.*0275***	*Smoking*	1.90 (1.03–3.40)	*0*.*0392*
**LUSC:**					
*H63D variant*	1.80 (0.59–5.24)	0.3084	C282Y *variant*	0.32 (0.13–0.78)	*0*.*0130*
*AgeAtDiagnosis*	1.00 (0.96–1.06)	0.6639	*AgeAtDiagnosis*	1.00 (0.97–1.06)	0.6231
*Tumor stage*	2.50 (1.10–5.68)	***0*.*0279***	*Tumor stage*	2.40 (1.08–5.51)	***0*.*0316***
*Smoking*	0.82 (0.36–1.84)	0.6286	*Smoking*	0.58 (0.24–1.42)	0.2349

LUAD (lung adenocarcinoma)

LUSC (lung squamous cell carcinoma)

HR (hazard ratio)

Covariates: Age at diagnosis (continuous), tumor stage (two categories, 3&4 vs 1&2), and smoking status (ever smoked vs never smoked)

**Fig 4:**
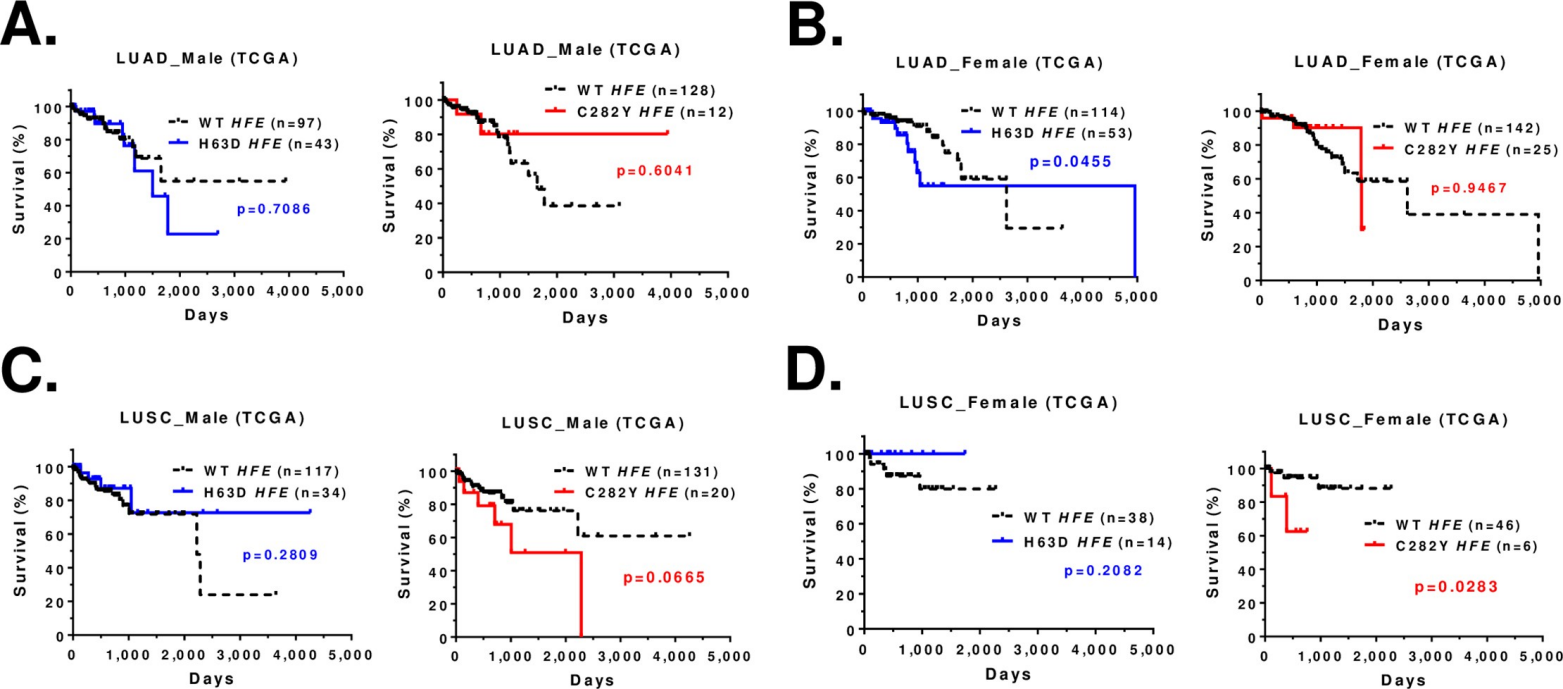
Kaplan-Meier survival curve for TCGA lung cancer patients (Caucasians) based on sex and *HFE* genotype. **(A)** Survival curve of male LUAD patients with WT or H63D *HFE* or C282Y *HFE*. **(B)** Survival curve of female LUAD patients with WT or H63D *HFE* or C282Y *HFE*. **(C)** Survival curve of male LUSC patients with WT or H63D *HFE* or C282Y *HFE*. **(D)** Survival curve of female LUSC patients with WT *HFE* or H63D *HFE* or C282Y *HFE*. Statistical analysis was performed by log-rank test and indicated as p value. Censored record is indicated as + in the graph.

When we stratify the survival data by sex and *HFE* genotype we found that male LUAD patients with H63D *HFE* had no survival difference compared to WT *HFE* and female LUAD patients with H63D *HFE* had significantly poorer survival compared to WT *HFE* (p = 0.0455) (**Fig 4A and 4B**). The dramatic difference in survival for male LUSC patients with C282Y *HFE* compared to WT *HFE* did not reach statistical significance (p = 0.0665); however, female LUSC patients with C282Y *HFE* had significantly poorer survival compared to WT *HFE* (p = 0.0283) (**Fig 4C and 4D**).

We further compared the survival of lung cancer patients based on *HFE* expression and sex. The survival pattern was not different regardless of *HFE* expression levels in lung cancer patients. There were no survival differences between lower 50% and upper 50%, or between lower 25% and upper 75% in TCGA lung cancer dataset (**S5 Fig**). In general, the survival curve between males and females was not different regardless of *HFE* expression levels in lung cancer patients (**S6 and S7 Figs**). However, when *HFE* expression level is in the upper 50%, male LUAD and LUSC patients tend to had poorer survival than females (**S6 and S7 Figs**).

## Discussion

The present study determined the frequency of *HFE* polymorphisms in lung cancer cell lines and human lung cancer patients. We found 7 of 10 lung cancer cell lines had the H63D or C282Y variant of the *HFE* gene. This result provided motivation to interrogate lung cancer samples from our institute and existing database such as TCGA for the frequency and impact of *HFE* gene variants on lung cancer.

There are significant *HFE* genotype and sex differences for survival and metastatic disease. In the TCGA lung cancer database, adenocarcinoma patients with H63D *HFE* trended to have poorer survival outcomes than WT *HFE*, but when the outcome data were stratified by sex the difference in survival outcome became statistically significant for females. The TCGA database does not include information on metastatic disease so we used our smaller internal database (PSHMC) and found a sex and genotype dependent effect for adenocarcinoma patients; males with the H63D *HFE* variant had less metastasis and females with the H63D *HFE* variant had more metastasis. There is no frequency increase in H63D *HFE* in the squamous cell cancer population or impact on the disease noted in our studies. These interesting results suggest that H63D *HFE* variant protects lung cancer metastasis in males, but enables metastasis in females. As far as we know, this is the first report for the impact of *HFE* variants and sex on lung cancer patients’ outcome.

In addition to the H63D variant of the *HFE* gene, the C282Y *HFE* variant is also found in the general population although at a lower prevalence. This gene variant has received considerable attention in hepatic cancers because of its tendency to be associated with hemochromatosis, the iron overload disease. In the TCGA database, individuals with squamous cell carcinoma that carry C282Y *HFE* have poorer survival than WT *HFE*. Moreover, when stratified for sex, it appears the females with C282Y are driving the significant difference as their survival outcome is much worse than males. There is no impact of this genotype of metastatic disease in the squamous cell cancer population. There is no statistically significant effect of C282Y *HFE* on the adenocarcinoma population, but there is a trend toward greater frequency of metastatic disease in males compared to females although the sample size is small. All these data suggest that females with C282Y *HFE* are a risk factor for lung squamous cell carcinoma. In our previous PSHMC GBM samples study, we found poorer survival in female metastatic brain tumor patients with C282Y *HFE* than WT *HFE* patients or male metastatic brain tumor patients with C282Y *HFE* [39]. The data implies that the metastatic potential of C282Y *HFE* is both cancer type and sex dependent.

Although we found *HFE* genotype and sex effect for lung cancer patient’s survival and metastatic disease, this is not related with the frequency of *HFE* genotype/alleles. For example, we observed higher frequency of C282Y *HFE* allele in female lung adenocarcinoma patients than male patients in TCGA; however, there was no survival difference between female C282Y *HFE* and male C282Y *HFE* lung adenocarcinoma patients. Instead, we found survival difference between female H63D *HFE* adenocarcinoma patients and female WT *HFE* adenocarcinoma patients.

In the present study, the level of *HFE* gene expression was not different between WT *HFE* and *HFE* variants of lung cancer patients (matched normal, primary tumors) in TCGA lung cancer data. In addition, *HFE* gene expression level did not impact survival of TCGA lung cancer patients (LUAD, LUSC) even when stratified for sex. These results suggest that poor survival in female lung adenocarcinoma with H63D *HFE* and female lung squamous cell cancer with C282Y *HFE* is not due to the expression level of *HFE* variants or frequency of *HFE* genotype but rather the function of *HFE* variant itself. There are many reports for the function of H63D or C282Y HFE in human cancer. For example, at the cellular level, the HFE H63D variant alters cholesterol metabolism [47], Endoplasmic Reticulum stress [48] and alters cancer phenotype [41, 49] including the protein profiles of exosomes [50]. Mouse H67D Hfe variant (human equivalent for H63D HFE variant) also alters macrophage function [51]. The C282Y HFE variant contributes therapy resistance and increased tumor burden [49]. Therefore, we will pursue the impact of sex and *HFE* variants in LUAD and LUSC in animal models as a future direction.

There are no consistent results between PSHMC and TCGA lung cancer samples for *HFE* genotype and cancer patient survival. When we compare the key variables between the TCGA lung cancer and the PSHMC samples, there are difference in age at diagnosis (e.g., median age: 70 years old for PSHMC vs. 67 years old for TCGA LUAD; p = 0.0026), smoking status (e.g., PSHMC vs. TCGA LUAD; p<0.0001), and the vital status (e.g., PSHMC vs. TCGA LUAD; p = 0.001) suggesting the PSHMC and TCGA cohorts have important distinctions. Therefore, we only showed Kaplan-Meier survival curve of PSHMC samples without Cox-regression results.

In summary, the H63D *HFE* gene variant is associated with poor survival and increased metastasis in female, but not male, LUAD patients. Although the frequency of C282Y *HFE* gene variant is higher in female LUAD patients than males, there was no gene effect on survival in these patients. However, the C282Y *HFE* gene variant is associated with poor survival in LUSC patients, especially females. The present findings indicate that there is a distinct impact of H63D and C282Y *HFE* variants in two different subtype of human lung cancers and impact of the genotype is influenced by sex.

## Supporting information

S1 Fig Association between *HFE* gene expression and *HFE* genotype or sex in TCGA LUAD patients. (A) *HFE* gene expression based on WT *HFE* vs. H63D *HFE* in matched normal or primary tumor LUAD patients. (B) *HFE* gene expression based on WT *HFE* vs. C282Y *HFE* in matched normal or primary tumor LUAD patients. (C) *HFE* gene expression based on males vs. females in matched normal or primary tumor LUAD patients. P value was calculated from Wilcoxon rank sum tests to compare *HFE* expression values in the *HFE* mutant vs. *HFE* wild type.
(PPTX)

S2 Fig Association between *HFE* gene expression and *HFE* genotype or sex in TCGA LUSC patients. (A) *HFE* gene expression based on WT *HFE* vs. H63D *HFE* in matched normal or primary tumor LUSC patients. (B) *HFE* gene expression based on WT *HFE* vs. C282Y *HFE* in matched normal or primary tumor LUSC patients. (C) *HFE* gene expression based on males vs. females in matched normal or primary tumor LUSC patients. P value was calculated from Wilcoxon rank sum tests to compare *HFE* expression values in the *HFE* mutant vs. *HFE* wild type.
(PPTX)

S3 Fig Kaplan-Meier survival curve for PSHMC lung cancer patients (Caucasians) with *HFE* genotype. (A) Survival curve of LUAD patients with WT *HFE* or H63D *HFE*. (B) Survival curve of LUAD patients with WT *HFE* or C282Y *HFE*. (C) Survival curve of LUSC patients with WT *HFE* or H63D *HFE*. (D) Survival curve of LUSC patients with WT *HFE* or C282Y *HFE*. Statistical analysis was performed by log-rank test and indicated as p value. Censored record is indicated as + in the graph.
(PPTX)

S4 Fig Kaplan-Meier survival curve for TCGA lung cancer patients (Caucasians) with *HFE* genotype. (A) Survival curve of LUAD patients with WT *HFE* or heterozygote or homozygote H63D *HFE*. (B) Survival curve of LUSC patients with WT *HFE* or heterozygote or homozygote C282Y *HFE*. LUSC with C282Y *HFE* heterozygote had poorer survival than WT *HFE* (p = 0.0052). Statistical analysis was performed by log-rank test and indicated as p value. Censored record is indicated as+ in the graph.
(PPTX)

S5 Fig Effect of *HFE* gene expression level on survival of TCGA lung cancer patients. (A) Kaplan-Meier survival curve of TCGA LUAD patients based on lower 50% or upper 50% of *HFE* gene expression. (B) Survival curve of TCGA LUAD patients between lower 25% and upper 75% of *HFE* gene expression. (C) Survival curve of TCGA LUSC patients based on lower 50% or upper 50% of *HFE* gene expression. (D) Survival curve of TCGA LUSC patients between lower 25% and upper 75% of *HFE* gene expression. P value was calculated from log rank tests to compare survival times in groups defined by *HFE* expression level.
(PPTX)

S6 Fig Effect of *HFE* gene expression level and sex on survival of TCGA LUAD data. Survival curve between males and females of TCGA LUAD patients based on *HFE* gene expression at lower 25% (A) or lower 50% (B) or upper 50% (C) or upper 75% (D). Log rank tests were used to compare survival times in groups defined by *HFE* expression level.
(PPTX)

S7 Fig Effect of *HFE* gene expression level and sex on survival of TCGA LUSC data. Survival curve between males and females of TCGA LUSC patients based on *HFE* gene expression at lower 25% (A) or lower 50% (B) or upper 50% (C) or upper 75% (D). Log rank tests were used to compare survival times in groups defined by *HFE* expression level.
(PPTX)

S1 Table Frequency of *HFE* genotype and alleles in Caucasian lung cancer patients at PSHMC and 1000Genome data. (DOCX)

S2 Table Frequency of *HFE* genotype and alleles in TCGA lung cancer (Caucasian) and 1000Genome. (DOCX)

S3 Table Frequency of *HFE* genotype and alleles based on sex of Caucasian lung cancer patients at PSHMC. (DOCX)

## Abbreviations

ALK: Anaplastic lymphoma kinase
ATCC: American Type Culture Collection
EGFR: Epidermal growth factor receptor
FGFR1: Fibroblast growth factor receptor 1
GBM: glioblastoma
KRAS: K-ras
MHC: major histocompatibility complex
LUAD: lung adenocarcinoma
LUSC: lung squamous cell carcinoma
NB: blood derived normal
NSCLC: non-small cell lung cancer
PSHMC: Penn State Hershey Medical Center
PTEN: Phosphatase and tensin homolog
RPMI: Roswell Park Memorial Institute
SCLC: small cell lung cancer
SLC11A1: Natural resistance-associated macrophage protein 1gene
SNP: Single Nucleotide Polymorphism
SNVs: Single Nucleotide Variants
TCGA: The Cancer Genome Atlas
TF: transferrin
TFRC: transferrin receptor
TP: primary solid tumor
WT: wild type
