# Supplementary material for: Impact of HFE variants and sex in lung cancer
Source: PLoS One. 2019 Dec 19;14(12):e0226821. doi: 10.1371/journal.pone.0226821 (PMC6922424; doi:10.1371/journal.pone.0226821)
Supplement: S2 Fig — (A) HFE gene expression based on WT HFE vs. H63D HFE in matched normal or primary tumor LUSC patients. (B) HFE gene expression based on WT HFE vs. C282Y HFE in matched normal or primary tumor LUSC patients. (C) HFE gene expression based on males vs. females in matched normal or primary tumor LUSC patients. P value was calculated from Wilcoxon rank sum tests to compare HFE expression values in the HFE mutant vs. HFE wild type. (PPTX) [file pone.0226821.s002.pptx]

## Slide 1
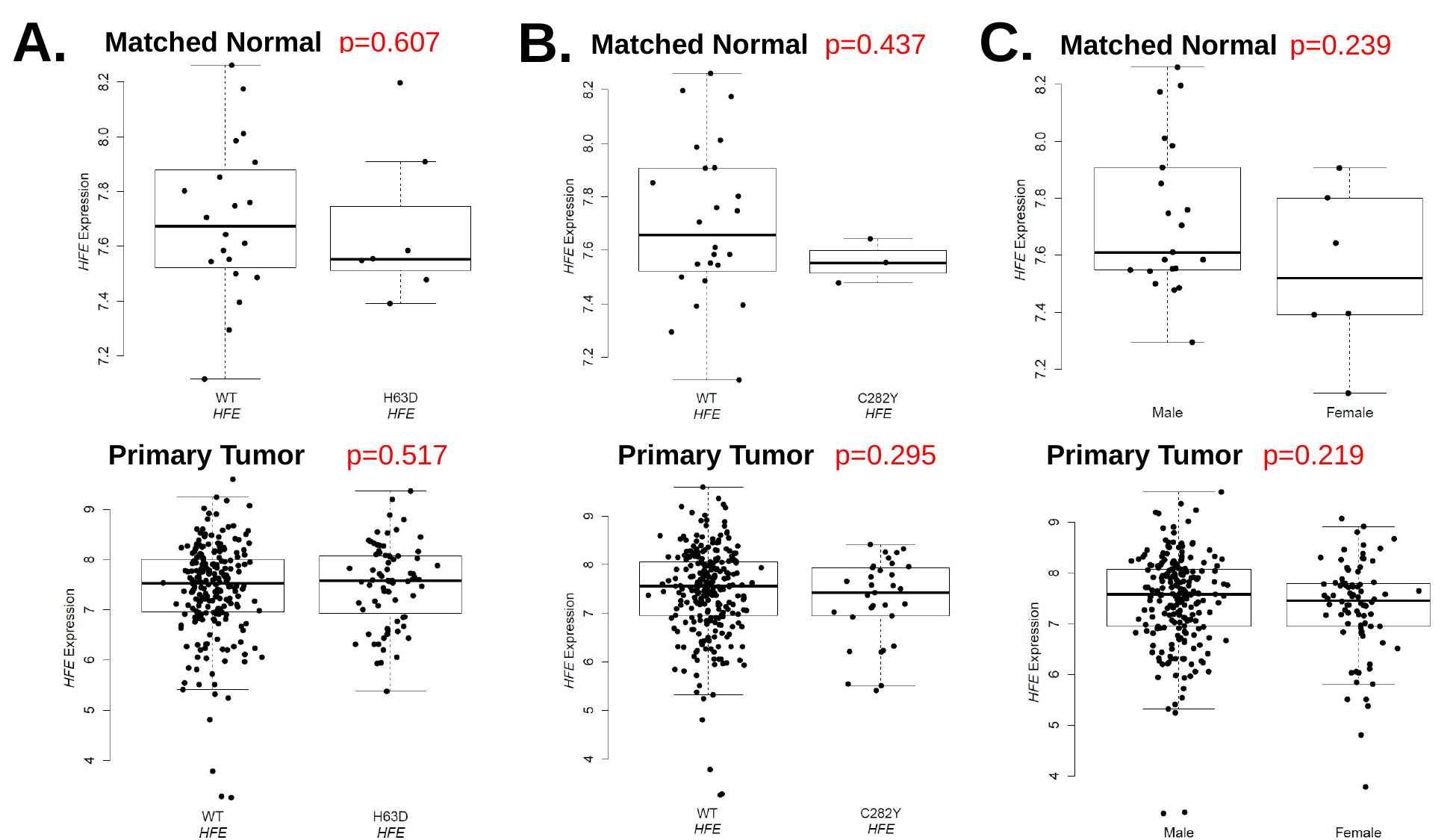

A.
C.
B.
Matched Normal
p=0.607
Matched Normal
p=0.437
Matched Normal
p=0.239
Primary Tumor
p=0.517
Primary Tumor
p=0.295
Primary Tumor
p=0.219
