# Supplementary figures and images for: Impact of HFE variants and sex in lung cancer
Source: PLoS One. 2019 Dec 19;14(12):e0226821. doi: 10.1371/journal.pone.0226821 (PMC6922424; doi:10.1371/journal.pone.0226821)

## Slide 1
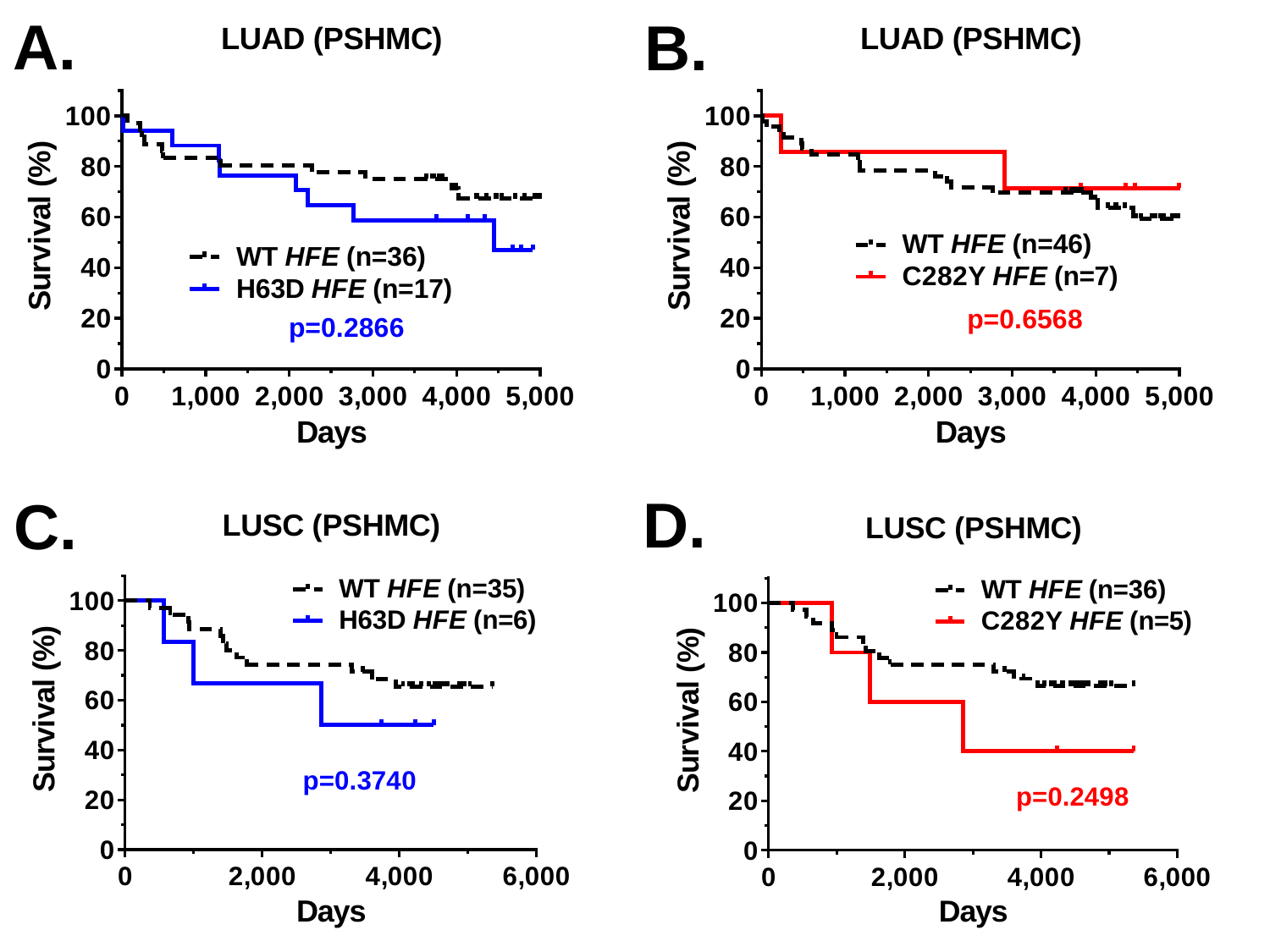

A.
B.
D.
C.

Supplement: S3 Fig — (A) Survival curve of LUAD patients with WT HFE or H63D HFE. (B) Survival curve of LUAD patients with WT HFE or C282Y HFE. (C) Survival curve of LUSC patients with WT HFE or H63D HFE. (D) Survival curve of LUSC patients with WT HFE or C282Y HFE. Statistical analysis was performed by log-rank test and indicated as p value. Censored record is indicated as + in the graph. (PPTX) [file pone.0226821.s003.pptx]

## Slide 1
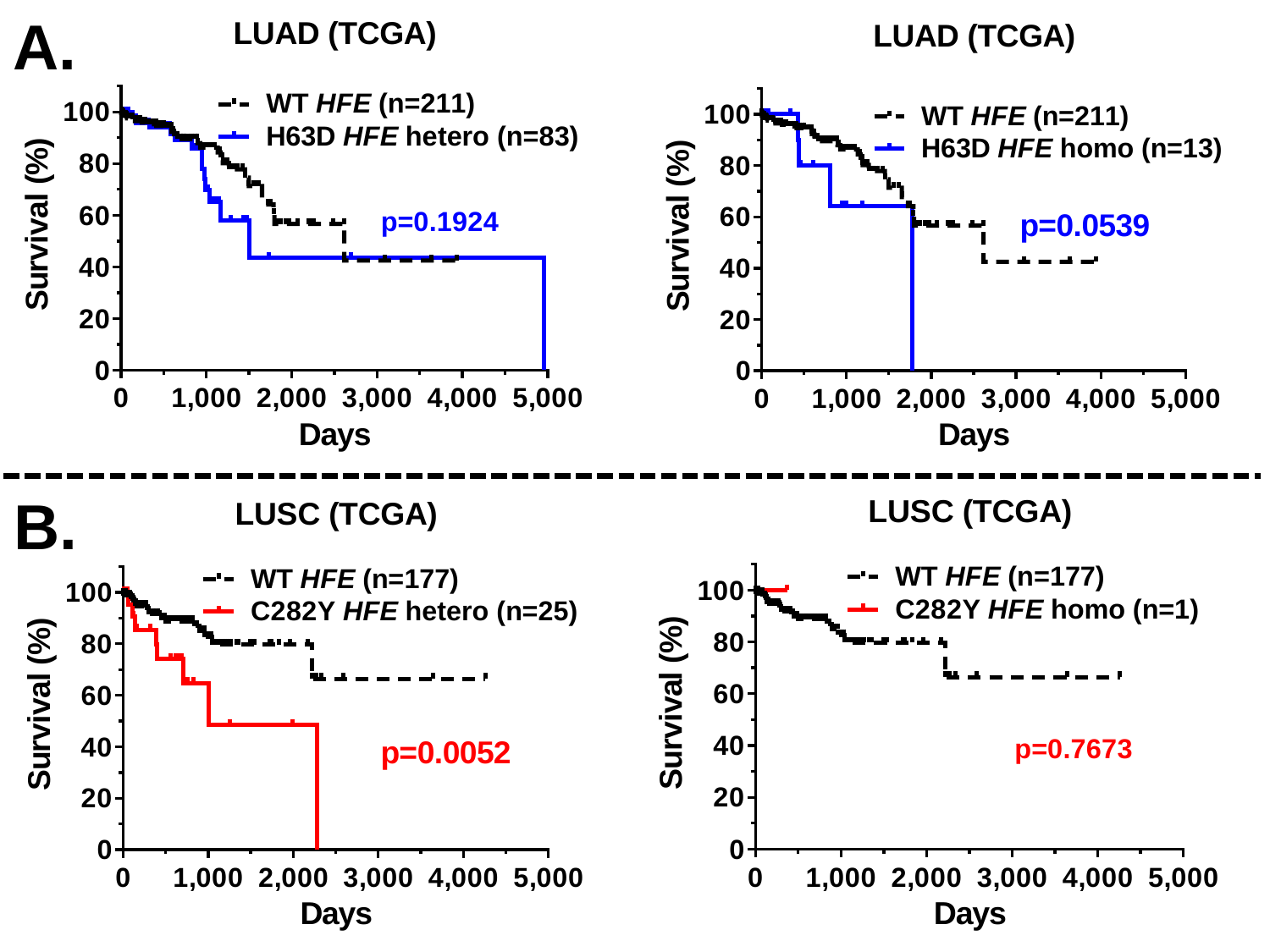

A.
B.

Supplement: S4 Fig — (A) Survival curve of LUAD patients with WT HFE or heterozygote or homozygote H63D HFE. (B) Survival curve of LUSC patients with WT HFE or heterozygote or homozygote C282Y HFE. LUSC with C282Y HFE heterozygote had poorer survival than WT HFE (p = 0.0052). Statistical analysis was performed by log-rank test and indicated as p value. Censored record is indicated as+ in the graph. (PPTX) [file pone.0226821.s004.pptx]

## Slide 1
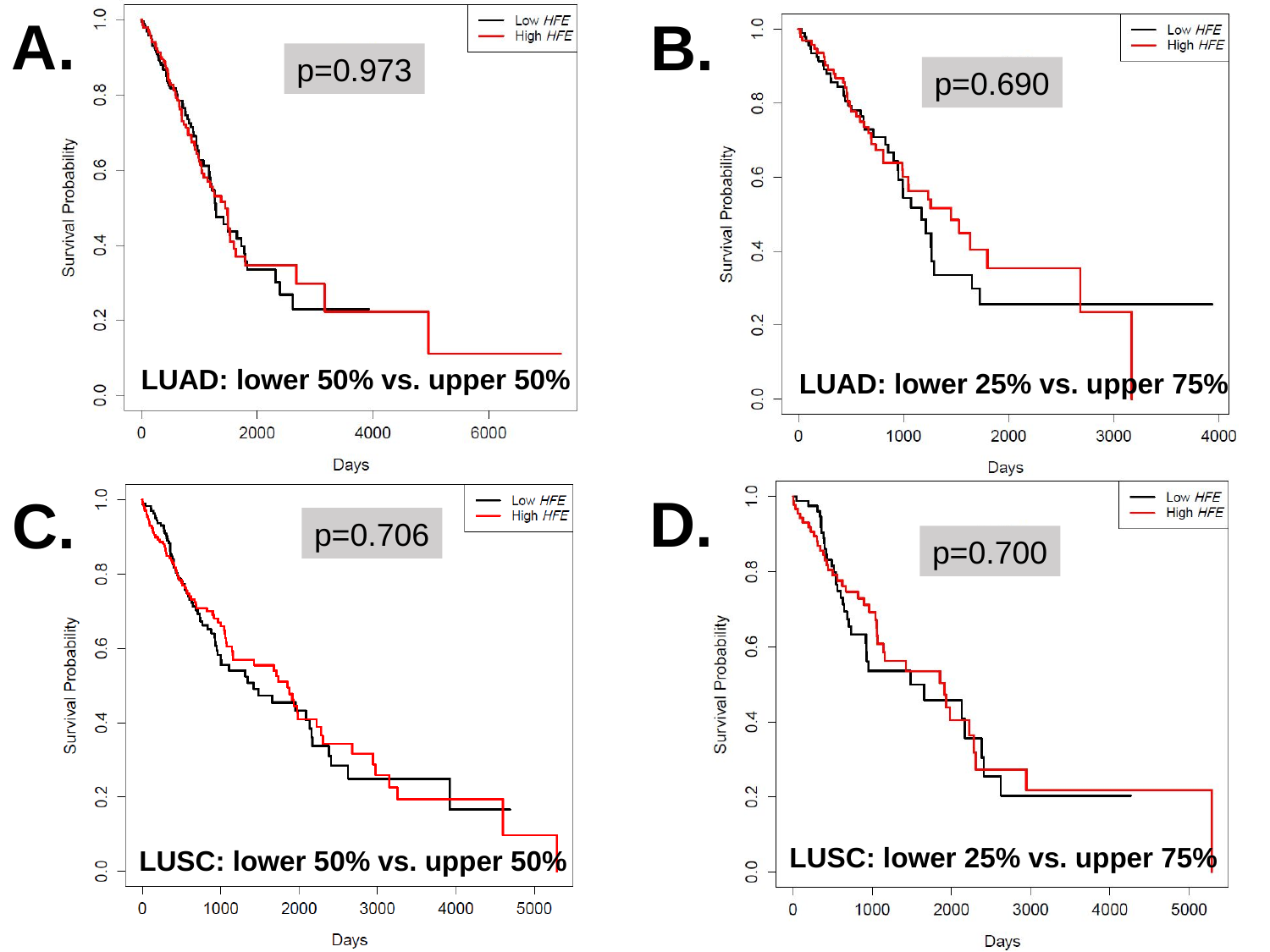

A.
B.
p=0.973
p=0.690
LUAD: lower 50% vs. upper 50%
LUAD: lower 25% vs. upper 75%
D.
C.
p=0.706
p=0.700
LUSC: lower 25% vs. upper 75%
LUSC: lower 50% vs. upper 50%

Supplement: S5 Fig — (A) Kaplan-Meier survival curve of TCGA LUAD patients based on lower 50% or upper 50% of HFE gene expression. (B) Survival curve of TCGA LUAD patients between lower 25% and upper 75% of HFE gene expression. (C) Survival curve of TCGA LUSC patients based on lower 50% or upper 50% of HFE gene expression. (D) Survival curve of TCGA LUSC patients between lower 25% and upper 75% of HFE gene expression. P value was calculated from log rank tests to compare survival times in groups defined by HFE expression level. (PPTX) [file pone.0226821.s005.pptx]

## Slide 1
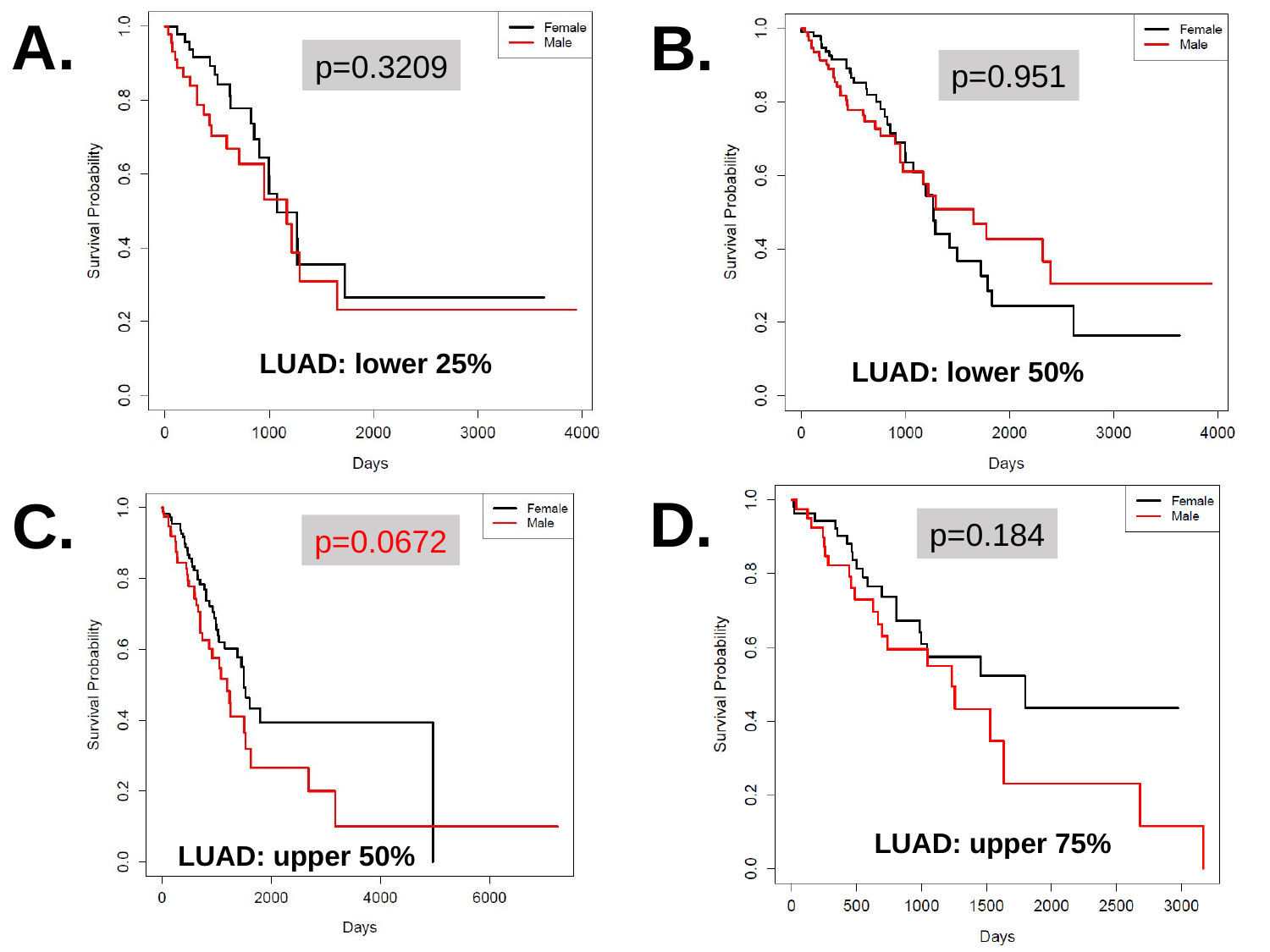

A.
B.
p=0.3209
p=0.951
LUAD: lower 25%
LUAD: lower 50%
D.
C.
p=0.184
p=0.0672
LUAD: upper 75%
LUAD: upper 50%

Supplement: S6 Fig — Survival curve between males and females of TCGA LUAD patients based on HFE gene expression at lower 25% (A) or lower 50% (B) or upper 50% (C) or upper 75% (D). Log rank tests were used to compare survival times in groups defined by HFE expression level. (PPTX) [file pone.0226821.s006.pptx]

## Slide 1
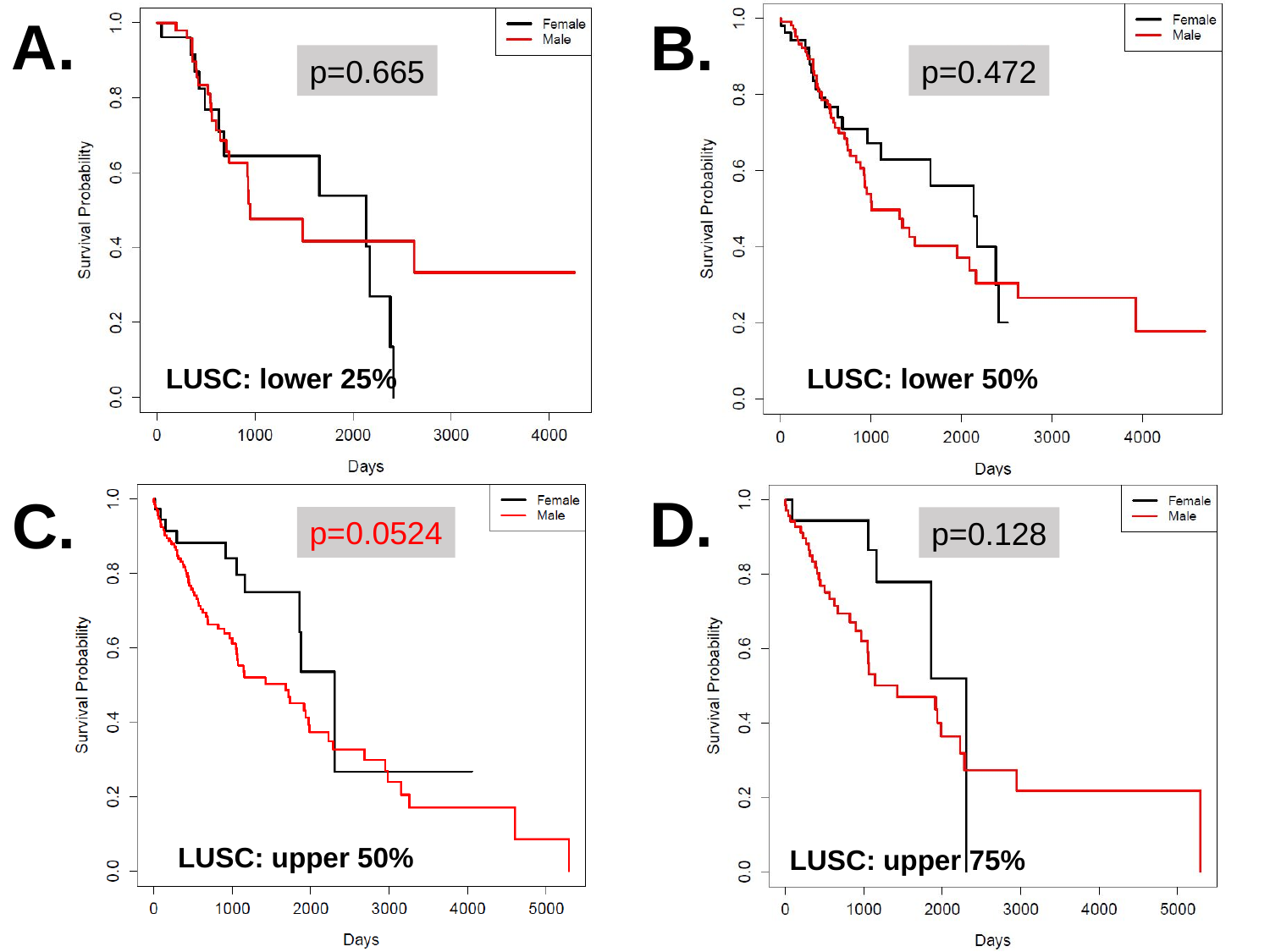

A.
B.
p=0.665
p=0.472
LUSC: lower 25%
LUSC: lower 50%
D.
C.
p=0.0524
p=0.128
LUSC: upper 50%
LUSC: upper 75%

Supplement: S7 Fig — Survival curve between males and females of TCGA LUSC patients based on HFE gene expression at lower 25% (A) or lower 50% (B) or upper 50% (C) or upper 75% (D). Log rank tests were used to compare survival times in groups defined by HFE expression level. (PPTX) [file pone.0226821.s007.pptx]
