# Supplementary material for: Impact of HFE variants and sex in lung cancer
Source: PLoS One. 2019 Dec 19;14(12):e0226821. doi: 10.1371/journal.pone.0226821 (PMC6922424; doi:10.1371/journal.pone.0226821)
Supplement: S1 Table — (DOCX) [file pone.0226821.s008.docx]

**S1 Table. Frequency of *HFE* genotype and alleles in Caucasian lung cancer patients at PSHMC and 1000Genome data.**

|  | **PSHMC LUAD (n=53)** | **PSHMC LUSC (n=41)** | **PSHMC non-cancer (n=94) ^a^** | **1000Genome Phase 3 (n=185) ^b^** |
| --- | --- | --- | --- | --- |
| **Genotype** |  |  |  |  |
| *H63/D63* (heterozygote) | 12/53 (22.6%) | 6/41 (14.6%) | 27/94 (28.7%) |  |
| *D63/D63* (homozygote) | 5/53 (9.4%) | 0/41 (0.0%) | 2/94 (2.1%) |  |
| *C282/Y282* (heterozygote) | 5/53 (9.4%) | 4/41 (9.8%) | 9/94 (9.6%) |  |
| *Y282/Y282* (homozygote) | 2/53 (3.8%) | 1/41 (2.4%) | 0 (0.0%) |  |
| **Alleles** |  |  |  |  |
| *H63D HFE* | 22/106 (20.8%) | 6/82 (7.3%) | 31/188 (16.5%) | (17.2%) |
| *C282Y HFE* | 9/106 (8.5%) | 6/82 (7.3%) | 9/188 (4.8%) | (4.3%) |
| **Fisher’s exact test** |  |  |  |  |
| LUAD vs. LUSC | **p=0.007 (*H63D HFE*)**  p=1.0 (*C282Y HFE*) | |  |  |
| LUAD or LUSC vs. PSHMC non-cancer | p= 0.4293 (*H63D HFE*)  p= 0.2137 (*C282Y HFE*) | **p= 0.0336 (*H63D HFE*)**  p= 0.3979 (*C282Y HFE*) |  |  |
| LUAD or LUSC vs. 1000Genome | p=0.18 (*H63D HFE*)  p=0.08 (*C282Y HFE*) | **p=0.04 (*H63D HFE*)**  p=0.24 (*C282Y HFE*) |  |  |

LUAD (lung adenocarcinoma)

LUSC (lung squamous cell carcinoma)

**^a^**Lee SY et al. PLoS One. 2017;12(3):e0174778.

**^b^**There are a total of 1,077 samples (527 male, 550 female) listed on the website, however, only a subset have sequences. There are 185 European subpopulation (80 male, 102 female, 3 unknown). Age information is not available.
