# Supplementary material for: Impact of HFE variants and sex in lung cancer
Source: PLoS One. 2019 Dec 19;14(12):e0226821. doi: 10.1371/journal.pone.0226821 (PMC6922424; doi:10.1371/journal.pone.0226821)
Supplement: S2 Table — (DOCX) [file pone.0226821.s009.docx]

**S2 Table. Frequency of *HFE* genotype and alleles in TCGA lung cancer (Caucasian) and 1000Genome.**

|  | **TCGA LUAD** | | **TCGA LUSC** | | **PSHMC non-cancer ^a^** | **1000Genome Phase 3 ^b^** |
| --- | --- | --- | --- | --- | --- | --- |
|  | **NB**  **(n= 307)** | **TP**  **(n= 381)** | **NB**  **(n= 203)** | **TP**  **(n= 300)** | **(n=94)** | **(n=185)** |
| **Genotype** |  |  |  |  |  |  |
| *H63/D63* (heterozygote) | 83 (27.0%) | 98 (25.7%) | 44 (21.7%) | 66 (22.0%) | 27/94 (69.1%) |  |
| *D63/D63* (homozygote) | 13 (4.2%) | 14 (3.7%) | 4 (2.0%) | 10 (3.3%) | 2/94 (2.1%) |  |
| *C282/Y282* (heterozygote) | 34 (11.1%) | 44 (11.5%) | 25 (12.3%) | 32 (10.7%) | 9/94 (9.6%) |  |
| *Y282/Y282* (homozygote) | 3 (1.0%) | 3 (0.8%) | 1 (0.5%) | 1 (0.3%) | 0 (0.0%) |  |
| **Alleles** |  |  |  |  |  |  |
| *H63D HFE* | 109/614 (17.8%) | 126/762 (16.5%) | 52/406 (12.8%) | 86/600 (14.3%) | 31/188 (16.5%) | (17.2%) |
| *C282Y HFE* | 40/614 (6.5%) | 50/762 (6.6%) | 27/406 (6.7%) | 34/600 (5.7%) | 9/188  (4.8%) | (4.3%) |
| **Fisher’s exact test** |  |  |  |  |  |  |
| LUAD or LUSC vs. PSHMC non-cancer | p= 0.8228 (*H63D HFE*)  p= 0.4864 (*C282Y HFE*) | p= 0.9114 (*H63D HFE*)  p= 0.4033 (*C282Y HFE*) | p= 0.1944 (*H63D HFE*)  p=0.4603 (*C282Y HFE*) | p= 0.3394 (*H63D HFE*)  p= 0.7159 (*C282Y HFE*) |  |  |
| LUAD or LUSC vs. 1000Genome | p=0.2303 (*H63D HFE*)  p=0.1201 (*C282Y HFE*) | p=0.5578 (*H63D HFE*)  p=0.1069 (*C282Y HFE*) | p=0.3116 (*H63D HFE*)  p=0.1171 (*C282Y HFE*) | p=0.6501 (*H63D HFE*)  p=0.3002 (*C282Y HFE*) |  |  |

Values were expressed as n=N (%)

LUAD (lung adenocarcinoma)

LUSC (lung squamous cell carcinoma)

NB (blood normal)

TP (tumor patient)

**^a^**Lee SY et al. PLoS One. 2017;12(3):e0174778. [40]

**^b^**There are a total of 1,077 samples (527 male, 550 female) listed on the website, however, only a subset have sequences. There are 185 European subpopulation (80 male, 102 female, 3 unknown). Age information is not available.
