# Supplementary material for: Impact of HFE variants and sex in lung cancer
Source: PLoS One. 2019 Dec 19;14(12):e0226821. doi: 10.1371/journal.pone.0226821 (PMC6922424; doi:10.1371/journal.pone.0226821)
Supplement: S3 Table — (DOCX) [file pone.0226821.s010.docx]

**S3 Table. Frequency of *HFE* genotype and alleles based on sex of Caucasian lung cancer patients at PSHMC.**

|  | **PSHMC LUAD (n=53)** | | **PSHMC LUSC (n=41)** | |
| --- | --- | --- | --- | --- |
|  | Male (n=23) | Female (n=30) | Male (n=28) | Female (n=13) |
| **Genotype** |  |  |  |  |
| *H63/D63* (heterozygote) | 6/23 (26.1%) | 6/30 (20.0%) | 4/28 (14.3%) | 2/13 (15.4%) |
| *D63/D63* (homozygote) | 3/23 (13.0%) | 2/30 (6.7%) | 0/28 (0.0%) | 0/13 (0.0%) |
| *C282/Y282* (heterozygote) | 3/23 (13.0%) | 2/30 (6.7%) | 4/28 (14.3%) | 0/13 (0.0%) |
| *Y282/Y282* (homozygote) | 1/23 (4.3%) | 1/30 (3.3%) | 0/28 (0.0%) | 1/13 (7.7%) |
| **Alleles** |  |  |  |  |
| *H63D HFE* | 12/46 (26.1%) | 10/60 (16.7%) | 4/56 (7.1%) | 2/26 (7.7%) |
| *C282Y HFE* | 5/46 (10.9%) | 4/60 (6.7%) | 4/56 (7.1%) | 2/26 (7.7%) |
| **Fisher’s exact test (Male vs. Female)** | p=0.224 (*H63D HFE*)  p=0.490 (*C282Y HFE*) | | p=1.0 (*H63D HFE*)  p=1.0 (*C282Y HFE*) | |

LUAD (lung adenocarcinoma)

LUSC (lung squamous cell carcinoma)
